# In This Issue

**Published:** 1996

## The Classification of Alcoholics: Typology Theories From the 19th Century to the Present

For over 150 years, physicians and researchers have attempted to categorize alcoholics according to various schemes or typologies. Dr. Thomas F. Babor recounts the history of alcoholism typologies through the prescientific period of clinical speculation, the Jellinek era of review and synthesis, and the post-Jellinek period of increasingly sophisticated empirical research. Dr. Babor concludes that although the typologies developed during each of these periods vary greatly with respect to their methodologies, the resulting alcoholism subtypes share enough characteristics to allow grouping them into two overarching categories. (pp. 6–14)

## Type I and Type II Alcoholism: An Update

Astudy of Swedish adoptees and their biological and adoptive parents resulted in the identification of two distinct alcoholism subtypes, type I and type II. These two subtypes differ according to the age at which alcoholism develops; the relative contributions of predisposing genetic and environmental factors; the gender and personality traits of the alcoholic; and whether co-occurring psychiatric disorders, such as antisocial behavior, are present. In this article, Drs. C. Robert Cloninger, Sören Sigvardsson, and Michael Bohman provide an update on the type I-type II typology and discuss findings from a recent study replicating the initial adoption study on which the typology is based. (pp. 18–23)

## Subtypes of Alcoholics Based on Psychometric Measures

One approach to subtyping alcoholics is using psychometric tests to assess the patients’ psychological characteristics, notes Dr. John P. Allen. The resulting typologies may potentially have meaningful implications for alcoholism treatment. Several tests have been used to identify alcoholic subtypes. These tests categorize alcoholics according to their personalities, their motivation for treatment, and the existence of co-occurring psychiatric disorders. Dr. Allen reviews typology schemes based on these characteristics, as well as a hybrid model that classifies alcoholics along various sets of criteria. Researchers have not yet evaluated sufficiently, however, how effective these typologies will be in selecting and planning the appropriate treatment strategy for each patient. (pp. 24–29)

## Type A and Type B Alcoholism: Applicability Across Subpopulations and Treatment Settings

Alcoholism is a complex disorder with many different causes and outcomes. Some studies suggest that all alcoholic subjects can be assigned to one of two types that differ consistently in several factors related to the causes, symptoms, and adverse consequences of alcohol use. Dr. Samuel A. Ball explores a model developed by researchers at the University of Connecticut in which so-called type B alcoholism appears to represent a more severe form of the disorder than does type A alcoholism. This type A-type B alcoholism typology, which is similar to Cloninger’s type I-type II, may help explain the different causes, courses, prognoses, and outcomes for the disorder. (pp. 30–35)

## Patient Placement Criteria: Linking Typologies to Managed Care

Most alcoholism typologies are based on differences in patient characteristics. However, an alternative approach, which was sparked by the managed care movement, focuses on variations in treatment service. With this approach, sets of criteria are developed to match patients to the appropriate treatment (e.g., outpatient versus inpatient treatment). The goal is to contain costs while ensuring the optimal allocation of resources. In this article, Dr. Leslie C. Morey describes one frequently used criteria set developed by the American Society for Addiction Medicine. These criteria evaluate patients along six dimensions before matching them with one of four levels of care. Dr. Morey reviews the advantages and disadvantages of these criteria and examines how patient placement criteria can be linked to other alcoholism typologies. (pp. 36–44)

## The Development of Alcoholic Subtypes: Risk Variation Among Alcoholic Families During the Early Childhood Years

Alcoholism frequently is associated with other psychiatric disorders, most commonly with antisocial personality disorder (ASPD). The presence of ASPD in an alcoholic parent also influences the child’s risk for developing alcoholism, according to Drs. Robert A. Zucker, Deborah A. Ellis, C. Raymond Bingham, and Hiram E. Fitzgerald. Such high-risk families frequently have a greater number of alcoholic relatives or a higher prevalence of other psychiatric disorders. Because of this association, researchers are using the presence or absence of ASPD to distinguish different subtypes of alcoholics. The authors review a long-term study that compares alcoholism risk factors among children of alcoholic men with and without ASPD and children of nonalcoholic parents. The study has identified significant differences among the three sets of parents, the children’s home environments, and the children’s behavior, which may influence whether a child develops alcoholism later in life. (pp. 46–54)

## Gender and Alcoholic Subtypes

Gender may be used as a distinction in alcoholism subtyping schemes. Some typologies, such as Cloninger’s type I-type II scheme, place female alcoholics in a single category (i.e., type I). Other typologies, including Babor and colleagues’ type A-type B scheme, are based on findings that women, like men, may differ in their risk for and severity of alcoholism. Drs. Francis K. Del Boca and Michie N. Hesselbrock analyzed a population of female and male alcoholics and determined that although those with severe or mild alcoholism differ little from each other in their personal profiles, men and women with a moderately severe form of the disease tend to be divided by sex according to their co-occurring psychopathologies (i.e., depression, anxiety, or antisocial personality). This finding has implications for determining the etiologies and treatment of men’s and women’s alcoholism. The authors caution, however, that conclusions drawn from one set of subjects cannot necessarily be extended to the general population. (pp. 56–62)

## Typology Research Questionnaires

Alcoholism typologies often are based on aspects of subjects’ personalities, their drinking patterns, and their family histories of alcoholism. To distinguish these characteristics, researchers often rely on personality questionnaires. In this section, Ms. Kathryn G. Ingle reviews five of the most common questionnaires used in alcoholism typology research: the Minnesota Multiphasic Personality Inventory (MMPI), the MacAndrew Alcoholism Scale (MAC), the Eysenck Personality Questionnaire (EPQ), the Tridimensional Personality Questionnaire (TPQ), and the Connecticut Typology Questionnaire. The development of each questionnaire is traced and examples of their questions and uses are provided. (pp. 63–66)

